# What We Owe to Experiments on Animals

**Published:** 1903-12-05

**Authors:** Stephen Paget


### Hospital Clinics and Medical Progress.

### WHAT WE OWE TO EXPERIMENTS ON ANIMALS.

By Stephen Paget.

The facts that are briefly stated in the following articles have already been published by the writer, at greater length, and with many more references, in a book called "Experiments on Animals." (New and Revised Edition. John Murray. 1903.) By the kindness of Mr. Murray, the writer has been allowed to give, in this series of articles, a short summary of the case that is presented more fully in the book. This summary is put in very simple words, because the articles are to be issued, when they are finished, as a cheap pamphlet that everybody can understand. Such a pamphlet, for general reading, may be useful to many people who wish to know what has been gained in physiology, pathology, medicine, and surgery, by the help of these experiments.

### *I.—Physiology.*

### I. The Circulation of the Blood.

Galen (born 131 a.d.), by experiment on animals, proved that the arteries contain blood, and not the "breath of life." He says, "Having tied the exposed arteries above and below, we opened them between the ligatures, and showed that they were indeed full of blood."

Realdus (1516–1557) discovered the circulation of the blood through the lungs, but not the general circulation through the body. He says, of the circulation through the lungs, "No man hitherto has noted it, or left it on record, though it is most worthy of the observation of all men. If you will look, not only in the dead body, but also in the living animal, you will always find this pulmonary vein full of blood. Verily I pray you, O candid reader, studious of authority, but more studious of truth, to make experiment on animals."

Harvey (1578–1657) published in 1628 his book "On the Movement of the Heart and of the Blood in Animals." He begins by saying that he had found the movement of the heart very hard to understand, even after many observations made on living animals; and he goes on, "At last, having daily used greater insight and diligence, *by frequent examination inside many and various living animals*, and many observations put together, I came to believe that I had succeeded, and had escaped and got out of this labyrinth, and had discovered what I desired, the movement and use of the heart and the arteries."

(It is true, of course, that Harvey did not make this discovery by the help of these experiments alone. In his extreme old age, he told Boyle that the arrangement of the valves of the veins had "invited him to imagine" that the blood was sent to the limbs through the arteries, and was returned through the veins. But it is one thing to be invited to imagine a fact, and another thing to prove it; and Harvey's own book shows, quite plainly, that experiments on animals were of the utmost help to him.)

Malpighi (1661) discovered the capillaries; before his time, it was supposed that the blood percolated through "blind porosities" in the tissues, like water soaking through earth or through a sponge. It has been said that anybody could discover the circulation of the blood, with a dead body and an injecting syringe; Malpighi tried these injections again and again, and failed; but he was able, with a microscope of two lenses, to see the capillaries in a dead frog, and then, in a living frog, to see the blood moving through them.

Hales (1726) by experiments on animals, measured the blood-pressure in the chief arteries.

John Hunter, from an experiment on the growth of a buck's antlers, discovered the collateral circulation. A few months later (December, 1785), now that he could put trust in the collateral circulation, he tied the artery in "Hunter's canal" for the cure of aneurysm.

Poiseuille (1828), the inventor of the mercurial gauge for the blood-pressure, measured the blood-pressure with far greater accuracy than Hales had been able to use; and Marey (1863), the inventor of the improved instrument for measuring the pulse, and of an instrument for measuring the blood-pressure within the cavities of the heart, extended Poiseuille's method from physiology to the practical study of the variations of the blood-pressure in health and in disease.

Since Marey, many observations have been made on the more minute variations of the blood-pressure, the relations between blood-pressure and secretion, the influences of the nervous system on the heart and the arteries, the automatism of the heart-beat, and the influence of gravitation on the distribution of the blood. The incessant shifting and adjustment of the whole volume of the blood are a very complex part of life; and Harvey's work solved only one out of many problems.

### II. The Lacteals.

Before Asellius (1622), though the lacteals had been seen now and again, yet they had not been understood. On July 22nd, 1622, while he was demonstrating the movement of the diaphragm in a dog, Asellius saw them, "as it were many threads, very thin and very white. Thinking at first sight that they were nerves, I did not greatly heed them. But soon I saw that I was wrong, for I bethought me that the nerves which belong to the intestines are distinct from these threads, and very different from them, and have a separate course. Wherefore, struck by the newness of the matter, I stopped for a time silent, while one way and another there came to my mind the controversies that occupy anatomists, as to the mesenteric veins and their use; which controversies are as full of quarrels as of words. When I had pulled myself together, to make experiment, taking a very sharp scalpel, I pierce one of the larger threads. Scarcely had I hit it off, when I see a white fluid running out, like milk or cream. At which sight, when I could not contain my joy, *I have found it*, I say like Archimedes."

Asellius failed to trace the further course of the lacteals. Their continuity with the thoracic duct, and the passage of their contents into the blood, were discovered by Jehan Pecquet (1650). Having killed an animal by the removal of its heart, he saw milky fluid like the fluid in the lacteals oozing from the cut end of a vein, and, in the living animal, he succeeded in finding the thoracic duct. "At last," he says, "by careful examination deep down along the sides of the dorsal vertebræ, a sort of whiteness, as of a lacteal vessel, catches my eyes. It lay in a sinuous course, close up against the spine. I was in doubt, for all my scrutiny, whether I had to do with a nerve or with a vessel. Therefore, I put a ligature a little below the clavicular veins; and then the flaccidity above the ligature, and the swelling of the distended duct below the ligature, absolutely resolved my doubt."

From the discovery of the lacteals, came the discovery of the whole lymphatic system, by Rudbeck and others. It is to be observed, that as the lacteals and the thoracic duct, unless they are at work, are empty and collapsed, they had been overlooked by all the great anatomists of the Renaissance.

*To be continued.*

